# The *i*nfluence of skin-to-skin contact on *C*ortical *A*ctivity during *P*ainful procedures in preter*m* *i*nfants in the *n*eonatal *i*ntensive care unit (iCAP mini): study protocol for a randomized control trial

**DOI:** 10.1186/s13063-022-06424-4

**Published:** 2022-06-20

**Authors:** Marsha Campbell-Yeo, Britney Benoit, Aaron Newman, Celeste Johnston, Tim Bardouille, Bonnie Stevens, Arlene Jiang

**Affiliations:** 1 School of Nursing, Faculty of Health, Dalhousie University and IWK Health, Halifax, NS Canada; 2 Rankin School of Nursing, St. Francis Xavier University, Antigonish, NS Canada; 3 Faculty of Science, Department of Psychology & Neuroscience, Dalhousie University, Halifax, NS Canada; 4 McGill University, Montreal, QC Canada; 5 Department of Physics & Atmospheric Science, Dalhousie University, Halifax, NS Canada; 6 Lawrence S Bloomberg Faculty of Nursing, University of Toronto and Child Health Evaluative Sciences Program, Research Institute, The Hospital for Sick Children (SickKids), Toronto, ON Canada; 7 Dalhousie University, Halifax, NS Canada

**Keywords:** Infant/neonate, Prematurity, Pain, Acute, Skin-to-skin contact, Sweet taste, EEG-evoked potentials, Cortical, Bio-behavioural assessment, RCT

## Abstract

**Background:**

Strong evidence suggests that maternal-infant skin-to-skin contact (SSC) is effective in reducing behavioural responses to pain. Given the multi-sensory benefits of SSC, it is highly likely that SSC provided during pain in early life may reduce pain-induced brain activity. The aim of this study is to examine the effect of SSC compared to 24% sucrose on pain-induced activity in the preterm infant brain during a medically required heel lance. Secondary objectives include determining (a) differences between behavioural pain response and noxious-related brain activity during heel lance and (b) rate of adverse events across groups.

**Methods:**

We will randomly assign 126 babies (32 to 36 completed weeks gestational age) admitted to the neonatal intensive care unit, and their mothers within the first seven days of age to receive (i) SSC plus sterile water and (ii) 24% oral sucrose. Each baby will receive a medically indicated heel lance, following a no treatment baseline period. The primary outcome is noxious-related brain activity measured using an electroencephalogram (EEG) pain-specific event-related potential. Secondary outcomes include pain intensity measured using a bio-behavioural infant pain assessment tool (Premature Infant Pain Profile-Revised) and rate of adverse events.

**Discussion:**

This will be the first clinical trial to compare the effect of SSC and 24% sucrose on pain-induced brain activity in the preterm infant brain during a clinical noxious stimulus, measured using EEG. Given the negative neurodevelopmental outcomes associated with unmanaged pain, it is imperative that preterm babies receive the most effective pain-reducing treatments to improve their health outcomes. Our findings will have important implications in informing optimal pain assessment and management in preterm infants.

**Trial registration:**

ClinicalTrials.gov NCT03745963. Registered on November 19, 2018.

## Administrative information

Note: the numbers in curly brackets in this protocol refer to SPIRIT checklist item numbers. The order of the items has been modified to group similar items (see http://www.equator-network.org/reporting-guidelines/spirit-2013-statement-defining-standard-protocol-items-for-clinical-trials/).

**Table:** 

**Title**	**The** ***i*nfluence of skin-to-skin contact on** ***C*ortical *A*ctivity during** ***P*ainful procedures in preter*m*** ***i*nfants in the** ***NI*CU (iCAP mini): study protocol for a randomized control trial**
Trial registration	NCT03745963 ClinicalTrials.gov Date of registration: November 19, 2018
Protocol version {3}	Version 1.0 (23OCT2018)
Funding {4}	Nova Scotia Health Research Foundation, Project #1,023,060 The funder, Nova Scotia Health Research Foundation will have no role in the collection, management, analysis, and interpretation of data; writing of the report; nor the decision to submit the report for publication
Author details {5a}	Marsha Campbell-Yeo School of Nursing, Faculty of Health Dalhousie University and IWK Health, Halifax, Nova Scotia, Canada Britney Benoit Rankin School of Nursing St. Francis Xavier University, Antigonish, Nova Scotia · Canada Aaron Newman Faculty of Science, Department of Psychology & Neuroscience, Dalhousie University, Halifax, Nova Scotia, Canada Celeste Johnston Professor Emerita, McGill University, Montreal, Quebec, Canada Tim Bardouille Department of Physics & Atmospheric Science Dalhousie University, Halifax Nova Scotia, Canada Bonnie Stevens Lawrence S Bloomberg Faculty of Nursing, University of Toronto and Child Health Evaluative Sciences Program, Research Institute, The Hospital for Sick Children (SickKids), Toronto, Ontario, Canada Arlene Jiang, Dalhousie University, Halifax, Nova Scotia, Canada
Name and contact information for the trial sponsor {5b}	Investigator sponsored Marsha Campbell-Yeo, School of Nursing, Dalhousie University and IWK Health, Halifax Nova Scotia, CANADA
Role of sponsor {5c}	Oversight and conduct of the study

## Background and rationale {6a}

Humans in the last trimester of gestation are assumed to be in a pain-free environment in the womb. However, one in every ten babies worldwide is born preterm and often spends weeks in the neonatal intensive care unit (NICU) where they will undergo numerous painful procedures as part of their routine care [1–5]. A recent systematic review of epidemiological studies reported that preterm infants underwent a daily average of 12 painful procedures, with the majority receiving no interventions to alleviate pain [6]. These findings are consistent with a Canadian survey carried out by members of this research team, which indicated that higher exposure to painful procedures and lack of pain-relieving interventions were the most frequent in the youngest and sickest neonates [7]. At one time it was believed that preterm infants did not experience pain, but two decades of behavioural observation studies [8–11], as well as recent studies using near-infrared spectroscopy (NIRS) [12, 13], electroencephalography (EEG) [14–17], and functional magnetic resonance imaging (fMRI) [18] to document cortical responses to noxious stimuli provides compelling evidence that infants as young as 25 weeks gestational age experience pain. In addition to immediate deleterious physiological effects and suffering, untreated early pain has been associated with long term consequences in children, including cognitive [19, 20] and language deficits [21], motor delays [22, 23], behavioural problems [24], and poor executive functioning [24–26]. In addition to outcomes assessed through standardized measures, imaging studies have demonstrated decreased frontal and parietal brain width [27], and altered diffusion measures and functional connectivity in the temporal lobes [28] of infants highly exposed to pain.

### Sweet tasting solutions and non-pharmacological pain management in the NICU

While pharmacological interventions may appear to be a logical solution to minimize pain, they can be inadequate, unavailable, or impractical for the most common procedures such as heel lance, venipuncture, intravenous insertion, or intramuscular injection [29–31]. This is of significant concern, as together these procedures make up 94% of tissue-breaking painful exposures experienced by infants in hospital [32]. As such, we and others have examined the effectiveness of alternative interventions such as sweet tasting solutions (sucrose [33] or glucose [34]) and non-pharmacological strategies (skin-to-skin contact [35] (SSC), often referred to as kangaroo care, which is the upright ventral holding of a diaper clad baby on the bare chest of a mother or alternate care provider), non-nutritive sucking (pacifier) [36], or swaddling (bundling in a blanket) [36] to reduce neonatal pain response. There is now strong evidence from Cochrane and other systematic reviews that, based on bio-behavioural measures (physiological, facial, or body responses), oral sucrose [33] and non-sucrose sweet tasting solutions [34], as well as SSC [35], are the most effective methods to reduce pain associated with commonly performed needle-related and tissue-breaking procedures in preterm infants in the NICU. Despite this evidence, there remains little understanding of the effect of these interventions on nociceptive (i.e. pain-related) responses in the neonatal brain. Specifically, whether interventions used in clinical practice lead to modulation or blunting of noxious pain-related brain responses, or solely depresses the expression of behavioural responses (e.g. facial actions). A recent systematic review including 74 human studies (*n* = 7049) [33] has shown that intra-oral sucrose (currently considered standard of care for hospitalized infants) effectively reduces bio-behavioural responses of preterm infants undergoing procedural pain. Although widely practised, there is some evidence that brings the analgesic efficacy of sucrose into question. In one study by Slater [15], reported in the Lancet, sucrose decreased bio-behavioural pain scores in infants undergoing heel lance when compared to a no treatment control, however, sucrose did not significantly reduce the amplitude of a Noxious-related event-related potential measured using EEG, suggesting that its effects may be sedative in nature rather than analgesic [15]. These findings support concerns raised by Abbott and Guy [37], who demonstrated using a rat pup model that while behavioural response to pain was similar between rats that were administered morphine and pentobarbital, only morphine blunted the nociceptive pain specific response, demonstrating the difficulty in discerning between sedation and analgesia using solely bio-behavioural measures.

## Objectives {7}

The *primary objective* of this study is to characterize the effect of SSC condition compared to 24% oral sucrose on noxious-evoked brain activity in the preterm infant brain during a clinically required heel lance procedure.

*Secondary objectives* are to determine (a) differentiation between bio-behavioural pain response and noxious-related brain activity elicited by clinical heel lance; (b) frequency of adverse events between interventions across groups; and (c) maternal acceptability of study participation.

### Hypotheses

#### Primary

Infants randomized to the SSC condition, when compared to infants randomized to the 24% oral sucrose condition, will demonstrate a lower amplitude pain-specific event-related potential during a clinical (heel lance) noxious stimulus.

#### Secondary

Infants randomized to the SSC condition, when compared to infants randomized to the 24% oral sucrose, will demonstrate (a) less differentiation between bio-behavioural pain response and noxious-evoked pain activity elicited by clinical (heel lance) noxious stimulus and (b) fewer adverse events (i.e. episodes of choking, apnea, bradycardia, temperature instability, need for repeat heel lance or “rescue sucrose” doses).

## Trial design {8}

A single centre, single-blind, parallel randomized controlled two-arm trial superiority design, similar to our previous studies in this population[38], will be utilized. Infants will be randomized to (i) SSC or (ii) 24% oral sucrose where they will undergo a clinically required heel lance. All aspects of the study will undergo institutional ethical approval. No infant will undergo pain associated with a heel lance procedure without intervention as per recent recommendations [39].

## Methods: participants, interventions, and outcomes

### Study setting {9}

The study will be conducted in the NICU of the IWK Health in Halifax, Nova Scotia, a 40-bed tertiary level referral unit.

### Eligibility criteria {10}

Infants considered to be medically stable, whose parents have provided informed consent, and require a clinically indicated heel lance, will be recruited to participate. Infants delivered between 32 and 36 completed weeks gestational age (GA) at birth admitted to NICU whose parents can read and write English will be approached for inclusion within the first seven days of age. Determination of stability will be made in consultation with the attending neonatal staff. Exclusion criteria are major congenital anomalies, received or receiving opioids in the previous 24 h, immediate post-op surgical period (< 72 h), history of hypoxic-ischemic encephalopathy requiring cooling, and contraindication for sucrose administration (e.g. unable to swallow, paralysis) due to safety concerns or inability to assess pain accurately.

### Who will take informed consent? {26a}

Consent for the research team to approach potentially eligible participants will be facilitated by the clinical team. A research nurse will determine eligibility, provide study information, and obtain signed informed consent.

### Additional consent provisions for collection and use of participant data and biological specimens {26b}

N/A: No biological specimens were collected as part of this trial.

## Interventions

### Explanation for the choice of comparators {6b}

Currently, the provision of 24% sucrose prior to a heel lance is considered standard of care and will be used as a comparator to SSC.

Twenty-four per cent sucrose (0.1 ml) will be delivered by a research nurse experienced in NICU care, onto the anterior surface of the tongue, commencing 2 min prior to data collection, doses may be repeated every 2 min throughout the painful procedure as deemed necessary by the clinical care provider [40]. The only modification to usual practice will be to position the infant to ensure optimal data readings from the EEG net and to visualize their face on the video recording, to enable scoring of pain responses.

### Intervention description {11a}

#### Intervention arm

Infants allocated to the SSC arm will be placed in upright, ventral SSC with their mother at least 15 min prior to data collection.

### Criteria for discontinuing or modifying allocated interventions {11b}

It is unlikely that there will be a need to modify the allocation intervention. However, parents may choose to withdraw their infant from the study for any reason at any time.

### Strategies to improve adherence to interventions {11c}

Every effort will be made to ensure the fidelity of the intervention. Any deviation will be documented using an intervention fidelity checklist completed by the research nurse at the end of the procedure. Any need for additional (e.g. rescue) pain management strategies will be monitored and compared monthly across groups.

### Relevant concomitant care permitted or prohibited during the trial {11d}

No other changes in clinical care will occur outside the study interventions.

### Provisions for post-trial care {30}

N/A: There is no post-trial care in this trial.

### Outcomes {12}

#### Noxious-related brain activity

Noxious-related brain activity will be measured from a pain-related event-related potential [14–16, 41] induced by a clinically required heel lance measured using a dense-array neonatal EEG recording. The primary outcome attributed to the clinically required heel lance will be a noxious-related N420-P560 event-related potential (ERP) which will specifically be examined and isolated at electrode Cz as previous research has reported pain-related activity at this site in both neonates [14, 16, 42] and adults [43].

The EEG data will be recorded using a 32 channel HydroCel Geodesic Sensor Net connected to a GES 400 EEG system (EGI, Eugene, OR, USA). Electrodes will be submerged for 5 min in a solution of warm water and infant shampoo. Electrode impendences will be checked to ensure they are below the impedance threshold of 100 kΩ. NetStation Acquisition software (version 5.2.0.2) will be used for recording of EEG activity from 0.5 to 30 Hz with a sampling rate of 1000 samples per second and a conversion of 24 bit. For all infants, regardless of randomization, data collection will begin with a continuous recording of a one-minute baseline of all outcome measures while the infant is resting in their incubator or cot (BL1). Following this, a non-noxious (NN1) control stimulus will be applied to the infant’s foot to capture a baseline response on EEG to a non-painful event. This non-noxious (sham) stimulus will consist of placing the heel lance against the foot and rotating it 90 degrees, so that when the lance is released it mimics the sensation of the heel lance procedure without the associated tissue-breaking and pain. The infant will then be placed in their assigned intervention group and will be re-exposed to the non-noxious control stimulus, followed by the clinical heel lance.

The method for time locking the non-noxious stimulus and noxious heel lance to the EEG recording in this study involves the use of audio-recording equipment to time-lock based on the audible spring-blade release of the heel lance. During both events, the lancet will be released. The sound of the heel lance release will be captured using a lapel microphone held by the research coordinator close to the infant’s foot, amplified through an USB audio interface (M-Audio Fast Track, M-Audio Inc., Cumberland, RI, USA), and linked to the EEG amplifier using an EGI Audio-Visual (AV) device through an AV device DIN adaptor and hypergrip cable (EGI, Eugene, OR, USA). This apparatus ensures that the sound associated with heel lance release is marked with millisecond precision on the continuous EEG recording. To test the accuracy of this time-locking method and ensure there is no latency through the audio-computer interface, the microphone input line and audio output line were connected to a digital oscilloscope and relative timing of the activity on both lines were observed during microphone activation. There was no appreciable latency between input and output, indicating a precise time-locking mechanism to isolate a pain-specific ERP.

#### Pain intensity

**Fig. 1:**
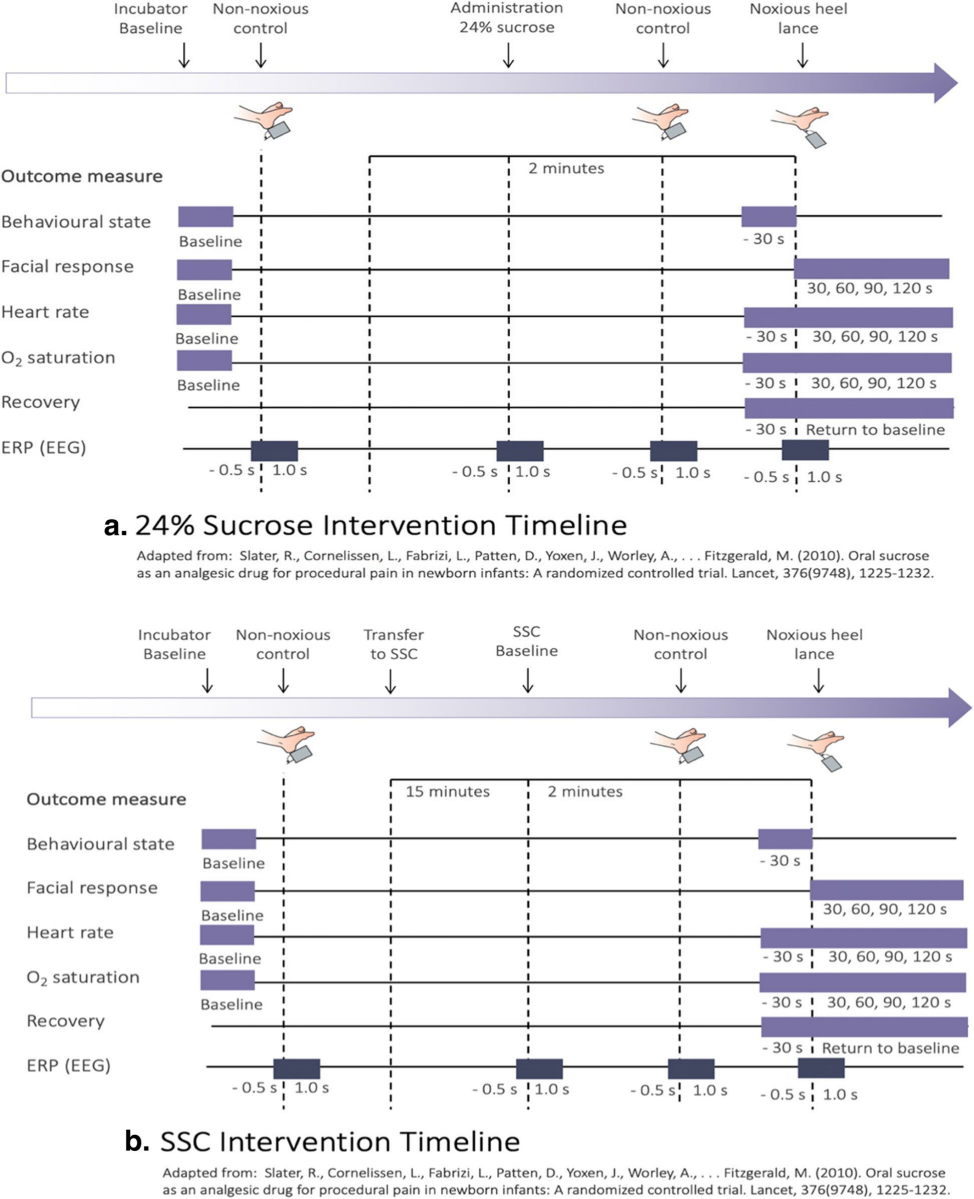
**a** Twenty-four per cent sucrose intervention timeline. **b** SCC intervention timeline

Pain intensity will be assessed using the Premature Infant Pain Profile—Revised (PIPP-R) [44, 45]. The original PIPP was developed by Stevens et al. in 1996 [46] and revised in 2012 [44]. The PIPP includes two physiological (heart rate, oxygen saturation), three behavioural (brow bulge, eye squeeze, nasolabial furrow), and two contextual (GA, behavioural state [BS]) variables known to modify pain responses. Each variable is scored on a four-point scale (0, 1, 2, 3) reflecting changes in magnitude from baseline values. GA and BS are reverse-scored to account for developmental and state differences in preterm and term infants abilities to respond to pain [46]. In the PIPP-R, the original seven variables of the PIPP are retained, but ordering and scoring of GA and BS has changed to ensure baseline characteristics prior to the painful event do not artificially inflate scores. The PIPP-R has demonstrated construct validation in infants of varying GAs during extreme group comparisons [44]. As facial actions are the most specific bio-behavioural indicator of acute procedural pain in newborn infants [47], the three individual facial actions within the PIPP-R will be analysed separately in addition to the composite PIPP-R score. Physiologic and behavioural data collection techniques, which have been developed and used extensively by this research team over the past decade, will be employed [48, 49]. For physiologic variables, heart rate and oxygen saturation will be measured continuously throughout the procedure via a pulse oximeter placed on the infant’s hand and recorded on the GES 400 EEG System 400 Physio 16 II. Behavioural state will be observed for 30 s prior to the noxious stimulus from video, and GA will be determined from the medical record. Time locking of the noxious-evoked stimulus and heel lance will be synchronized with EEG recordings, physiological data, and behavioural data using the time trigger device described previously. Electronic event markers will be used to synchronize all physiological and behavioural data, and to indicate four distinct phases of the standardized heel lance: (i) baseline data collection for 2 min; (ii) 2 min during and following sucrose administration; (iii) heel lance; and (iv) return to baseline for 2 min. Pain intensity will be calculated by trained coders, blinded to the study purpose and design, using newly developed facial coding software (Pain Assessment in Neonates, [PAiN]) developed recently by the Principal Investigator (PI) for previous research [38]. Inter-rater reliability of greater than 0.8 [50] with the trained coders for the PIPP-R scores will be established prior to data collection and monitored throughout the study using a random selection of infants after 20 infants are entered into the study. See Fig. 1 for overview of data collection procedures.

#### Adverse events and safety monitoring

As the HydroCel Geodesic Sensor Net requires that electrode sponges are soaked in a warm saline solution prior to application to the infant scalp, a thermal cap will be placed over the sensor net during data collection to reduce heat loss. Infant axillary temperature will be monitored every 15 min throughout data collection to ensure normothermia (36.5 to 37.5 °C). Infants will be monitored constantly during data collection (observation and continuous cardiorespiratory and oxygen saturation recording) in order to prevent any risk of skin irritation while wearing the HydroCel Geodesic Sensor Net as well any incidence of choking, apnea (defined as unexplained cessation of breathing for 20 s or longer, or a shorter respiratory pause associated with bradycardia, cyanosis, pallor, and/or hypotonia [51]), or bradycardia (defined as a 30 beat per minute drop from baseline, or when the heart rate is below 100 beats per minute). Adverse events, need for a repeat heel lance to allow for sufficient blood collection, or administration of additional doses of “rescue” sucrose will also be recorded on the tolerance/adverse events section of the intervention fidelity checklist, which has been developed and validated in previous research by the PI [38].

#### Demographic and background clinical data

Demographic and background clinical data characterizing the sample that will be collected from maternal and infant hospital charts will include: maternal age, maternal parity, maternal experience providing skin-to-skin contact to previous children, maternal medications, history of maternal diabetes during pregnancy, infant GA at birth, infant postnatal age at time of study participation, mode of delivery, infant birth weight, infant Apgar scores, infant sex, total number of previous painful procedures, primary diagnoses, severity of illness (Neonatal Therapeutic Interventions Scoring System [NTISS] [52], Score for Neonatal Acute Physiology-II [SNAP II] [53], and whether the infant has a history of receiving opioids.

### Participant timeline {13}

Participant timeline is shown in Fig. 1.

### Sample size {14}

Using a priori data [15], we will require 63 participants per group (total sample size of 126 for the two groups) to detect a 30% reduction in amplitude of the pain-specific ERP on EEG during the clinically required heel lance. The sample size calculation accounts for multiple testing across intervention groups and is based on a type I error probability of 5% and power of 80% [54]. Sixty-three participants per group also allows us to detect the smallest clinically important difference of one on the PIPP-R scale, with a standard deviation of two, considered a clinically relevant difference between the active interventions of SSC and 24% oral sucrose [55].

### Recruitment {15}

Recruitment is ongoing. Based on expected number of infants admitted to the NICU within the eligible gestational age range and recruitment rates from our previous neonatal pian studies we anticipate an expected enrollment of, on average, four participants monthly. Through daily communication with the clinical team, the research team will identity potentially eligible infants and parents. Parents of eligible infants will be approached by the research nurse; information will be provided, and consent obtained.

## Assignment of interventions: allocation

### Sequence generation {16a}

Participants will be randomized to groups using a password-protected REDCap database randomization tool. Sequence generation will be conducted using a computer-generated random number list know only to a consultant statistician not associated with the study and made available through the online portal. Randomization will be block-stratified by age (32 to < 34 weeks versus > or equal to 34 week to 36 completed weeks GA) to ensure the intervention groups are balanced with respect to GA. Intervention allocation concealment will be achieved by using randomly permuted blocks of two, four, and six [56] to minimize risk of allocation bias.

### Concealment mechanism {16b}

Allocation will be made available only at the time of randomization when the research personnel access the online portal. Intervention allocation concealment will be achieved by using randomly permuted blocks of two, four, and six [56] to minimize risk of allocation bias, unknown to the research team.

### Implementation {16c}

Following consent and prior to the medically indicated procedure, the research nurse will randomize the infant to obtain the study intervention allocation from the online portal. Once provided the information from the site, the research nurse will inform mothers of their study arm allocation and facilitate the timing of each infants’ clinically required heel lance with the clinical team.

## Assignment of interventions: Blinding

### Who will be blinded {17a}

In keeping with clinical trials where interventions cannot be blinded, we will ensure that data will be collected independently from those conducting analysis (blinded to group allocation) to minimize bias. Mothers, staff, and research nurses and assistants completing data collection will not be blinded to whether the infant has been randomly assigned to SSC condition or the 24% oral sucrose condition. All individuals carrying out data analysis will be blinded to group assignment. Additionally, EEG data analysts will have no prior knowledge of study groups and assignment. Close up video of the infants face during the painful procedure with no sound will be recorded to ensure that coders will be blinded to all aspects of group allocation and study purpose.

### Procedure for unblinding if needed {17b}

It is not anticipated that there will be a need to unblind the data prior to analyses.

## Data collection and management

### Plans for assessment and collection of outcomes {18a}

Set-up of EEG equipment, video recording, and physiological monitoring is anticipated to take 20–30 min. Baseline measurements (BL1 and BL2, 2 min each), stabilization in SSC after transfer (15 min), and heel lance procedure (three to 5 min) is estimated to take 20–25 min for a total data collection period of 40–60 min. Infants will be followed until completion of a regularly scheduled heel lance procedure.

### Plans to promote participant retention and complete follow-up {18b}

Follow-up is completed within minutes of finishing the procedure; therefore, it is unlikely there will be missing data due to loss to follow-up.

### Data management {19}

Collection of both bio-behavioural data and pain-specific ERPs on EEG have been previously demonstrated in clinical trials with this patient population [16, 57]. A challenge that may be encountered during data collection is loss of physiologic data (e.g. heart rate and electrophysiologic measurements) due to equipment failure or movement artefact. EEG procedures will be employed to control for and limit loss due to artefact. Research personnel experienced in EEG techniques will be present during data collection to monitor data recordings and make prompt adjustments in the instance of any recording malfunctions to limit data loss.

### Confidentiality {27}

Study participants will not be identified in any reports or publications of this research. Participant study data and videotapes will be kept in a locked file cabinet in a locked office at IWK Health. All data will have participant names removed and identified only by a code. The code will be kept in a locked file cabinet in a locked office at IWK Health. Study records will be kept for a minimum of 10 years. Only the staff involved in the research will see them. Members of the IWK Research Ethics Audit Committee or delegates of the Nova Scotia Health Research Foundation may look at the records to ensure the study is being conducted properly.

### Plans for collection, laboratory evaluation and storage of biological specimens for genetic or molecular analysis in this trial/future use {33}

N/A: No biological specimens were collected as part of this trial.

## Statistical methods

### Statistical methods for primary and secondary outcomes {20a}

Descriptive statistics will be used to summarize baseline characteristics using the Statistical Package for the Social Sciences (SPSS, IBM Corporation, Armonk, NY, USA). Student t tests and chi-square tests will be used, as appropriate, to assess potential differences across intervention groups at baseline.

#### Noxious-related brain activity

Data from the EEG recordings will be processed and statistically analysed using the MNE (v. 0.24.0) [58], and the event-related potential (ERP) Principal Component Analysis (PCA) Toolkit (v. 2.76) [59] in MATLAB (MathWorks Inc., Natick, MA, USA). For artefact detection and correction, a copy of the continuous EEG data will be bandpass filtered using MNE from 1 to 30 Hz using a zero-phase finite impulse response filter with a hamming window. Since Cz is provided as the default reference channel in the EGI system, all EEG data will be re-referenced to Fz (E17 in the EGI system). This will retain data recorded from Cz, an area with reported pain-related activity in both infants [14–16] and adults [43]. Fz has also previously used as the reference electrode in neonatal pain research [60].

This raw, continuous EEG data will then be segmented into epochs time-locked to the event markers of experimental interest (including noxious and non-noxious stimuli), ranging from 500 ms prior to each event marker to 1000 ms after, with each segment normalized by subtracting the mean amplitude value over the segment from each time point (effectively centering each segment at 0 µV). Epochs will be excluded if they contain movement artefacts on electrode Cz. Any epochs containing peak-to-peak signal amplitudes exceeding 150 µV will be considered contaminated with movement artifacts [60]. The data reduction technique of x (PCA [59] will be applied to the data at electrode Cz to identify mathematically independent components of the waveform. The resulting principal components (PCs) represent systematic variation in the amplitude of the EEG signal at different time points across the EEG tracing. The latency and amplitude of the generated principal components will be examined and the PC corresponding to a N420-P560 will be retained for subsequent phases of the analysis. Four principal components will be examined: two PCs for the SSC group (PC for non-noxious control stimulus, PC for noxious heel lance) and two PCs for the sucrose group (PC for non-noxious control stimulus, PC for noxious heel lance). Two-way analysis of variance (ANOVA) test will be run to assess the effect of stimulation type (non-noxious control stimulus, noxious heel lance) and treatment (SSC and sucrose) on the peak amplitude of the principal components. With this analysis, it is possible to assess if (1) exposure to heel lance produces a pain-specific component and (2) if the magnitude of this component varies between intervention groups [14, 61]. If there are identified differences between groups in any potential confounding variables (i.e. prior pain exposure, infant sex) analysis of covariance (ANCOVA) will be utilized to adjust for the potential effect of these covariates.

#### Pain intensity

An overall between-group comparison of the PIPP-R scores will be performed using an ANCOVA, where the covariate will be the GA stratification variable. If the overall comparison is significant at the 5% level, then the paired comparisons will be performed using a similar ANCOVA model. An appropriate multiple comparison Bonferroni procedure will be used to control the overall type I error probability. To conduct the subgroup analysis for GA, an interaction term will be added to the ANCOVA model. In each trial the binary adverse events variables will be compared using a multiple logistic model with intervention group and GA as independent variables. To control for multiplicity due to the number safety variables, a 5% significance will be used.

#### Adverse events

Mean frequency of occurrence of each of the safety surveillance outcomes will be compared across the groups using *t*-tests, with post hoc tests for intervention condition as appropriate.

### Interim analyses {21b}

One analysis of the primary outcome is planned at study end. There are no plans for an interim analysis.

### Methods for additional analyses (e.g. subgroup analyses) {20b}

A subgroup analysis based on GA will be conducted.

### Methods in analysis to handle protocol non-adherence and any statistical methods to handle missing data {20c}

It is not anticipated that there will be an issue with protocol non-adherence given that data are collected over a single intervention and that participants are infants.

### Plans to give access to the full protocol, participant level-data, and statistical code {31c}

The protocol will be freely available through an open access publication. There are no plans to have participant-level dataset, and statistical code readily available, however, individual requests will be considered.

## Oversight and monitoring

### Composition of the coordinating centre and trial steering committee {5d}

The PI will be responsible for the overall program of research. A 0.5 FTE Research Coordinator will be hired for the day-to-day management of the trial including study personnel, under the supervision of the PI.

The PI, Co-I’s, and Research Coordinator will comprise the RCT Steering Committee. The Steering Committee will meet bi-monthly in year one, quarterly thereafter in person and via teleconference, and at the end of the study to discuss results and dissemination.

### Composition of the data monitoring committee, its role and reporting structure {21a}

A data safety monitoring committee (DSMC) will be established consisting of three individuals (with expertise in neonatology, neuroscience, or biostatistics), independent of the investigators who have no financial, scientific, or other conflicts of interest with the trial.

### Adverse event reporting and harms {22}

The collection, assessment, and reporting of adverse events and other unintended effects of trial interventions or trial conduct will be done in accordance with Good Clinical Guideline Practices and Research Ethics Board (REB) approvals.

### Frequency and plans for auditing trial conduct {23}

All study documents will be kept in accordance with Good Clinical Guideline Practices and REB approvals. There are no planned audits, however, institutional auditing of clinical trials independent from investigators occur routinely.

### Plans for communicating important protocol amendments to relevant parties (e.g. trial participants, ethical committees) {25}

If any important protocol modifications (e.g. changes to eligibility criteria, outcomes, analyses) are required, communication of these changes will made through online portals (i.e. ethics, ClincialTrials.gov.), or email reports will be forwarded to relevant parties (e.g. investigators, REB, trial participants, trial registries, journals, regulators).

### Dissemination plans {31a}

Knowledge translation initiatives will be ongoing throughout the research process, with initial emphasis on raising healthcare professional and public awareness and interest in newborn pain management followed by increased knowledge and changing behaviours through updating of relevant policies and practices. Regular updates on the progress of the study will be provided to health professionals and managers on the participating unit via face-to-face meetings. When results from the study become available, focus will also be placed on targeting researchers, healthcare providers, administrators, and decision makers with new knowledge to inform newborn pain assessment and management practices, as well as subsequent research in the field. Three reports (1, 3, and 10 pages) will be generated for key clinical and policy stakeholders to disseminate the findings of the study. These reports will be disseminated nationally and internationally through existing affiliations with the Canadian Neonatal Network, Canadian Association of Pediatric Health Centres, Canadian Pediatric Society, both Canadian and International councils of Neonatal Nurses, Vermont Oxford Neonatal Network, and Nordic Pain in Early Life Association. Traditional end of grant knowledge translation initiatives targeting researchers and knowledge users will include publication in high impact academic peer-reviewed journals (e.g. Pediatrics, JAMA Pediatrics, PAIN, Birth), presentation at national and international conferences (e.g. International Symposium on Pediatric Pain, Canadian Pain Society Annual Scientific Meeting, Pediatric Academic Society, Council of International Neonatal Nurses), and presentations and webinars to key clinical decision makers and healthcare providers in the clinical setting locally and worldwide (e.g. Family Centered Care Council, National Canadian Premature Baby Association and March of Dimes). Our team has a strong online presence and will share study results as appropriate over social media platforms including Facebook and Twitter, as well as press releases to raise widespread public, healthcare provider, and parent awareness. Collectively, our team has contributed to practice change in our local institutions and worldwide through clinical practice guidelines. The PI and several members of the team regularly give public presentations (e.g. CIHR Café Scientifique) and disseminate research findings through print, radio, and television media. A YouTube video produced by the PI for parents to reduce pain in newborns has been viewed > 165,000 times in > 152 countries and was awarded first place in the inaugural CIHR Video Talks Competition. Moreover, the Centre for Pediatric Pain Research (CPPR), where the PI is based, has successfully reached millions of parents and care providers through the CIHR funded “It Doesn’t Have to Hurt” initiative in partnership with YummyMummyClub.ca to improve pain care for children.

## Discussion

To the best of our knowledge, we will be the first sufficiently powered randomized clinical trial examining the effect of SSC provided during a medically indicated heel lance on pain induced activity in the preterm brain compared to the provision of 24% sucrose.

### Why might maternal-infant skin-to-skin contact (SSC) be different?

Tactile perception is one of the first senses of fetal development, emerging at approximately seven to eight weeks gestation [62]. Thus, all newborns have the ability to perceive physical touch and demonstrate positive responses to stroking or massage [63]. Human infants have been shown to demonstrate reduced behavioural stress and improved sleep following gentle human touch [64, 65] and massage [66, 67]. Additionally, animal models of neonatal touch have demonstrated increases in endorphins, oxytocin, and serotonin—hormones that have been associated with modulating pain response [68–71]. While no studies to date have examined the neural mechanisms underlying the analgesic effect of touch on pain responses in infants, there is evidence to suggest that simple touch modulates nociceptive specific responses in adults [72]. When A delta and C nociceptors were selectively activated by high power laser pulses in healthy adults, application of tactile stimulation significantly reduced cortical laser-evoked potentials [72] and laser blink reflex (which is generated by brain stem circuits before nociceptive signals reach the cortex) [72]. This suggests a blunting of both cortical and sub-cortical nociceptive responses secondary to tactile stimulation.

Evidence related to SSC demonstrates significant benefit such as stabilization of physiologic parameters (e.g. temperature, heart, and respiratory rates), decreased occurrence of apneas, improved weight gain and growth, and accelerated maturation of autonomic and circadian systems [63, 73–77]. Related to this proposal, SSC has demonstrated benefit as an effective pain-relieving intervention for infants undergoing needle-related procedural pain [78]. In a recent update of our Cochrane systematic review and meta-analysis, including 25 studies (*n* = 2001 infants), SSC significantly reduced composite pain scores measured by the PIPP [46] at 30 (*n* = 268, MD − 3.21, 95% CI − 3.94 to − 2.48), 60 (*n* = 164, MD − 1.85, 95% CI − 3.03 to − 0.68), and 90 (*n* = 163, MD − 1.34, 95% CI − 2.56 to − 0.13) s following painful procedures. Of note, while holding a clothed baby provides some comfort, it appears that direct SSC is significantly more effective [79]. The underlying mechanisms of SSC are thought to be associated with blunting of sympathetic nervous system responses and up-regulation of parasympathetic nervous system responses through physiological neural regulators [80]. Specifically, SSC may elicit an inborn tactile receptor response that regulates vagal tone and release of endogenous opiates, oxytocin, and beta-endorphins [81, 82]. This proposed mechanism of action differs significantly from the primary gustatory mechanism of sweet tasting solutions. As such, it is highly plausible that SSC may inhibit nociceptive responses in the preterm brain [60, 83].

### Summary and justification for the proposed research

While there is strong evidence of the effectiveness of oral sucrose and SSC to reduce bio-behavioural responses to pain, based on the current standard of assessment, optimal pain management in the NICU is precluded by our lack of understanding of the effect of these interventions on (a) noxious pain-related responses in the neonatal brain, (b) the efficacy of SSC when compared to sucrose on noxious pain-related brain activity, and (c) the relationship between noxious-evoked brain activity and bio-behavioural responses to noxious stimuli in preterm infants. The proposed research study will address these significant gaps. The results of the study may help increase awareness and inform behaviour change and practice regarding the importance of parent presence and engagement in pain care in the NICU.

## Trial status

The trial is currently in the recruitment phase. Recruitment began on November 19, 2018 and is estimated to be completed on September 30, 2022. Protocol Version 1.0 (23OCT2018).

## Data Availability

The Principal Investigator and study team will have access to the final trial dataset.

## Abbreviations

ANOVA: Analysis of variance
ANCOVA: Analysis of covariance
BS: Behavioural state
CPPR: Centre for Pediatric Pain Research
DSMC: Data Safety Monitoring Committee
EEG: Electroencephalography
ERP: Event-related potential
fMRI: Functional magnetic resonance imaging
GA: Gestational age
iCAP mini: The influence of skin-to-skin contact on Cortical Activity during Painful procedures in preterm infants in the neonatal intensive care unit
mN: Millinewtons
NTISS: Neonatal Therapeutic Interventions Scoring System
NICU: Neonatal intensive care unit
NIRS: Near-infrared spectroscopy
PAiN: Pain Assessment in Neonates
PCA: Principal Component Analysis
PCs: Principal components
PI: Principal Investigator
PIPP: Premature Infant Pain Profile
PIPP-R: Premature Infant Pain Profile Revised
REB: Research Ethics Board
SNAP II: Score for Neonatal Acute Physiology-II
SSC: Skin-to-skin contact
